# Dual role of *BCL11B* in T-cell malignancies

**DOI:** 10.1097/BS9.0000000000000204

**Published:** 2024-09-17

**Authors:** Grzegorz K. Przybylski, Julia Przybylska, Yangqiu Li

**Affiliations:** a Institute of Human Genetics, Polish Academy of Sciences, Poznań, Poland; b Department of Rheumatology, Independent Public Health Care Facility, Międzychód, Poland; c Key Laboratory for Regenerative Medicine of Ministry of Education, Institute of Hematology, School of Medicine, Jinan University, Guangzhou, China.

**Keywords:** *BCL11B*, BER, *CTIP2*, Rit1, T-cell malignancy, T-ALL, TCL

## Abstract

The zinc finger transcription factor B-cell CLL/lymphoma 11B gene (*BCL11B*, *CTIP2*) plays a crucial role in T-cell development, but its role in T-cell malignancies has not yet been definitively clarified. In the literature, 2 contradictory hypotheses on the function of *BCL11B* exist. One suggests that *BCL11B* functions as tumor suppressor gene, and the other suggests that *BCL11B* functions as oncogene. The aim of this review is to revise the current knowledge about the function of *BCL11B* in T-cell malignancies, confront these 2 hypotheses and present a new model of dual role of *BCL11B* in T-cell malignancies and potential new therapeutic approach, based on recent findings of the function of *BCL11B* in DNA damage repair. Decreased *BCL11B* expression, resulting in deficient DNA repair, may facilitate DNA mutations in rapidly proliferating T-cell progenitors that undergo gene rearrangements, thereby leading to malignant transformation. On the other hand, decreased *BCL11B* expression and inefficient DNA repair may result in accumulation of DNA damages in genes crucial for the cell survival and in apoptosis of malignant T cells. We hypothesize that T-cell malignancies expressing high levels of *BCL11B* might be dependent on it. In those cases, targeted inhibition of *BCL11B* expression may have a therapeutic effect. The antitumor effect of *BCL11B* suppression might be strengthened by generation of induced T to NK cells (ITNK). Therefore, there is an urgent need to develop a specific *BCL11B* inhibitor.

## 1. INTRODUCTION

The C(2)H(2) zinc finger transcription factors B-cell CLL/lymphoma 11A (Bcl11a), encoded by the *BCL11A (CTIP1*) gene, and its paralog Bcl11b encoded by the *BCL11B (CTIP2*) gene, were first identified in mice in 2000, as binding partners of the chicken ovalbumin upstream promoter-transcription factor (COUP-TF) family of nuclear hormone receptors.^1^ One year later, human homologs of *BCL11A* and *BCL11B* were identified, and localized to chr2p13 and chr14q32.1, respectively.^2^ Despite substantial similarities in their structure and binding sites,^2–4^ the 2 factors have distinct biological functions and roles in lymphocyte development. Bcl11a has a broader function in hematopoiesis. It is responsible for the development of B cells but is also involved in the development of dendritic cells and switch from γ- to β-globin expression during the fetal to adult erythropoiesis transition.^4–7^ In contrast to *BCL11A,* and to its misleading name, *BCL11B* is neither expressed in B-cell CLL nor in B-cell lymphoma. In the hematopoietic system, *BCL11B* is expressed almost exclusively in the T-cell lineage. It is essential for α/β T lymphocytes development; *BCL11B* knockout mice are born without α/β T cells and die shortly after birth.^8–12^ *BCL11B* is crucial for the initial pro-T-cell commitment, positive selection and survival of double-positive thymocytes, lineage choice in postselection thymocytes and correct responses to developmental checkpoints.^13–15^ It is also responsible for proper function of mature T lymphocytes, and removal of Bcl11b at the double-positive stage of T-cell development or in T(reg) cells causes autoimmune diseases.^16,17^ Besides T cells, *BCL111B* is implicated in the development of other nonhematopoietic tissues, including neurogenesis, skin development, adipogenesis, tooth formation, and cranial suture ossification,^18,19^ but this is beyond the scope of this article.

Although in the last 2 decades many studies have been published on *BCL11B*, its role in T-cell malignancies has not yet been definitively clarified. The aim of this review is to discuss the 2 contradictory hypotheses on the function of BCL11B in T-cell neoplasms; tumor suppressor; or oncogene.

## 2. *BCL11B* ALTERATIONS IN T-CELL MALIGNANCIES

First recurrent chromosomal alteration involving *BCL11B*, t(5;14)(q35;q32), associated with expression of the *TLX3* oncogene, due its juxtaposition to *BCL11B*, were reported by Bernard et al.^20^ First rearrangement affecting directly the *BCL11B* gene was reported by Przybylski et al.^21^ This rearrangement resulted in the expression of the 5′ part of *BCL11B*, including exons 1 to 3, fused to the constant region of the T-cell receptor delta gene (*TRDC*). Since then, many other genetic alterations have been described in T-cell and T/myeloid-mixed phenotype acute leukemia.^22^ In some cases *BCL11B* was affected itself, resulting in its overexpression, or the expression of chimeric fusion transcripts (*ZEB2-BCL11B* fusion), but more frequently, transcriptionally active enhancer sequences, located close to *BCL11B*, affected the expression of other genes (*HOXA*, *NKX2-1*, and *NKX2-5*), translocated to it.^22–25^ The effect of chromosomal rearrangements activating known oncogenes is quite evident, but the effect of heterogenous rearrangements affecting *BCL11B* gene itself is less obvious, and has not yet been sufficiently studied.

## 3. *BCL11B* AS TUMOR SUPPRESSOR GENE

Originally, *BCL11B* was reported by the team of Kominami, as radiation-induced tumor suppressor gene (*Rit1*).^26^ Using genome-wide allelic loss analysis, it has been shown that in murine γ-ray-induced thymic lymphomas a region of chromosome 12, containing, at that time, unknown *BCL11B* gene, was frequently deleted. In subsequent studies, they have found bi-allelic changes of *BCL11B* in p53-proficient lymphomas, what suggested an association between the presence of functional p53 and inactivation of *BCL11B* in the lymphoma development.^27^ Furthermore, introduction of *BCL11B* into HeLa cells lacking *BCL11B* expression suppressed cell growth. The authors concluded that loss-of-function mutations of *BCL11B* contribute to cancer development. On the other hand, the same group showed that mice born with homozygous *BCL111B* knockout show block of α/β T-cell differentiation and die but do not develop T-cell malignancies.^12^ In a large study on T-ALL, monoallelic *BCL11B* deletions or missense mutations were found in 9% (10 of 117) of cases.^28^ Some of them disrupted the structure of zinc finger domains required for DNA binding. The authors postulated that *BCL11B* is a haploinsufficient tumor suppressor that collaborates with all major T-ALL oncogenic lesions in human thymocyte transformation, although this has not been confirmed in functional studies.

## 4. *BCL11B* AS ONCOGENE

In ATM^−/−^ mice heterozygous loss of *BCL11B* reduced lethal thymic lymphoma by suppressing lymphoma progression but not initiation. The suppression was associated with a T cell–mediated immune response, revealing a haploid insufficient function of Bcl11b in immune modulation against lymphoma and offering an explanation for the complex relationship between Bcl11b status with T-ALL prognosis.^29^ In our recent study, we created mice with heterozygous *BCL11B* deletion. These mice have a normal life span and do not develop malignancies.^30^ The hypothesis of the oncogene function of *BCL11B* is further supported by research of our group, showing that *BCL11B* is overexpressed in the majority of T-cell acute lymphoblastic leukemia (T-ALL),^21^ and inhibition of *BCL11B* using siRNA leads to apoptosis of malignant but not normal T cells.^31^ This indicates that malignant T cells need *BCL11B* for their survival. Subsequently, we showed that forced overexpression of *BCL11B* resulted in markedly increased resistance to radiomimetic drugs, whereas no influence on death-receptor apoptotic pathway was observed.^32^ Apoptosis resistance triggered by *BCL11B* overexpression was accompanied by a cell cycle delay caused by accumulation of cells at G1. This cell cycle restriction was associated with upregulation of cyclin-dependent kinase inhibitors. Moreover, the SKP2 gene encoding a protein of the ubiquitin-binding complex responsible for their degradation was repressed and the expression of the MYCN oncogene was silenced. Furthermore, it was shown that enhancer hijacking drives oncogenic *BCL11B* expression in lineage-ambiguous stem cell leukemia with expression of myeloid and T lymphoid markers. This upregulation was driven by chromosomal rearrangements that juxtapose *BCL11B* to superenhancers active in hematopoietic progenitors, or amplifications that generate a superenhancer from a noncoding elements distal to *BCL11B*. These data support the role of *BCL11B* overexpression as an oncogenic event in leukemia with T-cell markers.^33,34^ Our studies in human naïve T cells showed increased proliferation upon *BCL11B* overexpression and reduced proliferation upon its downregulation.^35^ The data suggest a potential role of *BCL11B* in tumor survival and encourage developing Bcl11b-inhibitory approaches as a potential tool to specifically target chemoresistant tumor cells. Very recently, we have showed that *BCL11B* promotes T-ALL cell survival via the XRCC5/C11ORF21 axis.^36^ These results are in line with our recent data showing better prognosis for patients with T-cell leukemia and lymphoma with *BCL11B* mutations.^37,38^ However, this research included a small number of cases with *BCL11B* mutations, and has to be confirmed in a larger study.

## 5. ROLE OF *BCL11B* IN DNA REPAIR

Very recent study on *BCL11B* involvement in DNA repair provided new important information on role in tumor development. Vickridge et al^39^ had shown that *BCL11B* increases the enzymatic activity of NTHL1 glycosylase and Pol β polymerase, responsible for base excision repair (BER) of DNA, by stimulating binding to their substrate. Furthermore, they showed that *BCL11B* knockdown increases DNA damage, delays the repair of oxidized bases and abasic sites, and increases the spontaneous and radiation-induced mutation rates, thereby leading to apoptosis of malignant cells. Ectopic overexpression of a small, lacking transcription regulation potential, fragment of *BCL11B (BCL11B*^*213-560*^) accelerated DNA repair and increased resistance to oxidative DNA damage. Most interestingly, they showed that overexpressed *BCL11B*^*213-560*^ cooperates with RAS oncogene in primary cell transformation, by repairing DNA damage caused by RAS induced production of reactive oxygen species, and thereby avoiding cellular senescence. The evidence for tumor suppressor or oncogene role of BCL11B has been summarized in Table 1.

**Table 1.** Evidence for tumor suppressor or oncogene role of *BCL11B.*

Source of evidence	Tumor suppressor	Oncogene
Chromosomal rearrangements involving *BCL11B*^20–25^	Rearrangements disrupting *BCL11B* in T-ALL	Rearrangements activating *BCL11B* in T-ALL
*BCL11B* inactivating mutations^26–28^	Increased rate of oncogenic mutations in T-ALL Development of radiation-induced T-cell lymphomas	Lead to apoptosis due to increased rate of mutations in genes necessary for cell survival
Homozygous *BCL11B* knockout mice^12^ Heterozygous *BCL11B* knockout mice^29,30^		Block of α/β T-cell differentiation, but no oncogenesis Do not develop cancer In ATM^−/−^ mice heterozygous loss of *BCL11B* reduces lethal thymic lymphoma
*BCL11B* suppression ^31,35,39^		Inhibition of *BCL11B* using siRNA leads to apoptosis of malignant but not normal T cells Higher susceptibility to chemotherapeutics
*BCL11B* overexpression ^21,32–36,39^	Introduction of *BCL11B* into HeLa cells lacking BCL11B expression suppressed cell growth	Increased resistance to chemotherapeutics Frequent in T-ALL Overexpressed *BCL11B* cooperates with RAS oncogene in primary cell transformation

## 6. THERAPEUTIC IMPLICATIONS OF THE DUAL ROLE OF *BCL11B* IN T-CELL MALIGNANCIES

**Figure 1.**
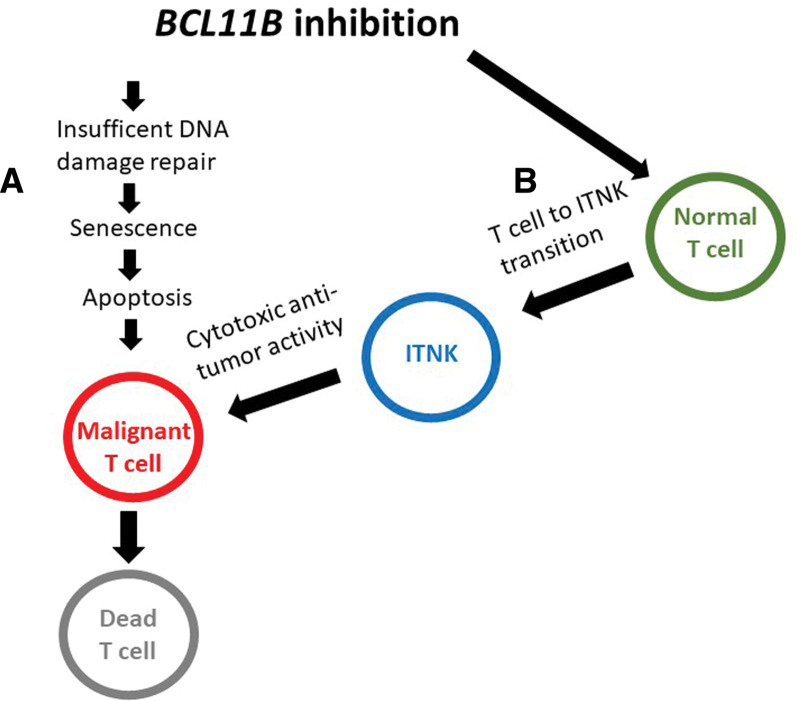
Potential therapeutic effect of *BCL11B* inhibition in T-cell malignancies. Suppression of *BCL11B* in malignant T cells results in insufficient DNA repair and thereby to cell senescence and apoptosis.^31,35^ Additionally, suppression of *BCL11B* in normal T cells leads to their transition to induced T to NK cells (ITNK) with cytotoxic antitumor activity.^9^ Both mechanisms synergistically contribute to the death of tumor cells.

Although *BCL11B* inactivation plays a role in accumulation of mutations and may be responsible for malignant transformation of T-cell progenitors, in already developed T-cell malignancy it is *BCL11B* expression that is necessary for the survival of malignant T cells and progression of the disease. This applies only to T-cell leukemia and lymphoma, and probably also to some nonhematopoietic tumors, that express high levels of *BCL11B.* Those malignant cells are dependent on DNA repair provided by *BCL11B*, and deprived of this would eventually undergo cell senescence and die. This makes inhibition of *BCL11B* a new, promising therapeutic approach, in patients with T-cell malignancies expressing high levels of *BCL11B*. Furthermore, Li et al^9^ had shown that *BCL11B* suppression in normal T lymphocytes leads to their transition to induced T to NK cells (ITNK) exhibiting a strong antitumor effect. Therefore, simultaneous inhibiting of *BCL11B* in malignant and normal T cells could have a synergistic antitumor effect (Fig. 1). To test the usefulness of *BCL11B* targeted therapy, a specific *BCL11B* inhibitor has to be developed.

## 7. SUMMARY

Early studies on *BCL11B* role in T-cell malignancies, mostly based on the deletions and mutations, suggested its tumor suppressor function. Later, more and more evidence was accumulated indicating that *BCL11B* might act as oncogene. Currently, based on recent findings of the function of *BCL11B* in base excision repair, it seems that *BCL11B* has a dual role in *BCL11B* malignancies. On the one hand, decreased *BCL11B* expression resulting in inefficient DNA repair, especially in rapidly proliferating and undergoing T-cell receptor genes rearrangements T-cell progenitors, may facilitate DNA lesions and lead to malignant transformation. On the other hand, decreased *BCL11B* expression and inefficient DNA repair may result in further accumulation of DNA damages in genes crucial for the cell survival, and result in apoptosis of malignant T cells.

It can be hypothesized that a fraction of T-cell malignancies, most likely those expressing high levels of *BCL11B*, might be dependent on it. In those cases inhibition of *BCL11B* might be a promising therapeutic approach, especially if generated by this ITNK cells will show antitumor activity.

## ACKNOWLEDGMENTS

This work was supported in part by the National Centre for Research and Development, Poland (No. WPC/BCL/2019) and the Intergovernmental International Cooperation on Scientific and Technological Innovation Project of Chinese Ministry of Science and Technology (No.2017YFE0131600).

Conflict of interest: The authors declare that they have no conflict of interest.
